# An Optimized Convolutional Neural Network for the 3D Point-Cloud Compression

**DOI:** 10.3390/s23042250

**Published:** 2023-02-16

**Authors:** Guoliang Luo, Bingqin He, Yanbo Xiong, Luqi Wang, Hui Wang, Zhiliang Zhu, Xiangren Shi

**Affiliations:** 1 Virtual Reality and Interactive Techniques Institute, East China Jiaotong University, Nanchang 330013, China; 2 School of Informatics, Xiamen University, Xiamen 361005, China

**Keywords:** point-cloud compression, convolutional neural network, activation function

## Abstract

Due to the tremendous volume taken by the 3D point-cloud models, knowing how to achieve the balance between a high compression ratio, a low distortion rate, and computing cost in point-cloud compression is a significant issue in the field of virtual reality (VR). Convolutional neural networks have been used in numerous point-cloud compression research approaches during the past few years in an effort to progress the research state. In this work, we have evaluated the effects of different network parameters, including neural network depth, stride, and activation function on point-cloud compression, resulting in an optimized convolutional neural network for compression. We first have analyzed earlier research on point-cloud compression based on convolutional neural networks before designing our own convolutional neural network. Then, we have modified our model parameters using the experimental data to further enhance the effect of point-cloud compression. Based on the experimental results, we have found that the neural network with the 4 layers and 2 strides parameter configuration using the *Sigmoid* activation function outperforms the default configuration by 208% in terms of the compression-distortion rate. The experimental results show that our findings are effective and universal and make a great contribution to the research of point-cloud compression using convolutional neural networks.

## 1. Introduction

A 3D point-cloud model is a collection of 3D data points in 3D space that represent a 3D shape or object. 3D point-cloud compression technology is also known as 6-dimensional data compression technology since point-cloud data are composed of geometric coordinates (X, Y, and Z) as well as RGB or other three color descriptors. Point-cloud data comes from a computer-aided design model, radar scanning, and depth camera acquisition. For example, Popișter et al. studied the point-cloud acquisition process with the help of a contact/non-contact 3D scanner and storage of point cloud data [1]. At present, there rarely exists such large bandwidth to support the direct transmission of point clouds on the network layer without compression. Therefore, it is necessary to compress point clouds. In the spatial domain, the point-cloud model typically contains a few hundreds and thousands to tens of millions of points. Without compression, the transmission rate of 30 frames per second for a point-cloud model with one million points per frame consumes a total bandwidth of 3.6 GB per second, placing strain on the available storage space and network transmission bandwidth. Consequently, attaining a low bit rate and low distortion point-cloud compression with limited storage space capacity and network transmission bandwidth remains a critical practical role in achieving efficient visualization on VR applications.

For the 3D point-cloud compression, the traditional octree-based point-cloud compression method in which the deeper depth will lead to exponential growth of data volume and the shallow depth will lose a lot of information. With the rapid development of convolutional neural networks, researchers proposed the method of using convolutional neural network to compress point clouds. For example, some researchers replaced the 2D-convolution in image compression with the 3D-convolution adapted to point clouds and converted the original point cloud into a voxel grid. This method led to a better compression result but had problems of low compression rate and high distortion rate. Therefore, we have conducted a number of experiments such as choosing the appropriate network parameters and activation functions to optimize the effect of compressing point clouds.

Convolutional neural networks require a large amount of computation and storage requirements. Thereby, knowing how to reduce computational costs and storage space is the focus of research on convolutional neural networks. Zhao et al. improved the performance and efficiency of 1-bit CNNs by combining Bayesian learning in CNN species 1-bit CNNs [2]. The computational redundancy of the complete model can be reduced within the allowed accuracy by pruning the neural network. Li et al. proposed the EagleEye pruning algorithm, which improved the efficiency and accuracy of pruning by the adaptive batch normalization of the network [3]. Zhang developed a new deep learning model: MCNs to reduce the storage cost of convolutional filters [4]. Similarly, Yeom et al. segmented a large number of experimental data using a SegNet-based CNN, greatly reducing the human time required to analyze the data when training the model [5]. Qayyum et al. evaluated each pre-trained convolutional neural network model for image classification processing, which provided a basis for reducing human subjectivity and time-consuming redundancy [6]. Due to improve the performance of related deep learning models, Wang et al. classified point-cloud data by focusing on the relationship between points and its neighbors, which achieved the purpose of capturing high-resolution or fine-grained features [7]. Liu et al. proposed a point-voxel CNN for 3D deep learning by pooling the advantages of voxels and points, which effectively improved the execution efficiency and effectiveness of convolution [8]. Xu et al. developed a deep learning model for point-cloud processing. By using the POEM method to build a 1-bit fully connected layer (Bi-FC) in point-cloud networks, the storage and computing costs of point-cloud data are effectively reduced [9]. Our work also benefits from these studies on increasing the performance of neural networks with the help of several activation functions. Specifically, Alkhouly et al. conducted comprehensive research on and the classification of common activation functions, which provided an in-depth analysis of the effects of several common activation functions on deep network architectures [10]. Liu et al. proposed an extended GLIT method for asymmetric activation functions, which expanded the range of activation functions and provided the possibility for further research on neural networks [11]. Kumar et al. explored a NewSigmoid function in neural networks, which is also as powerful as *tansig* and *logsig* [12]. Siegel et al. advanced the existing results on the approximation rates obtained for two layer neural networks with an increasing number of neurons [13]. These results were extended to polynomially decaying activation functions and to general bounded ones.

In this work, we have investigated the various effects of the neural network’s parameters, including stride, depth, and activation function on the impact of point-cloud compression. Based on the experimental results, we have designed an optimized scheme to maximize the compression performance. We first design the experimental scheme based on the convolutional neural network and then conduct experiments and modify our model parameters based on the experimental data to further optimize the point-cloud compression effect. Finally, we present a convolutional neural network that shows improved performance on the compression of 3D point cloud data.

This work contains the following major contributions:

- First, we have evaluated the compression effect by altering the parameters of depth, stride, and activation function of the neural networks. The experimental results show that the *Sigmoid* function outperforms the other activation functions.
- Second, we have proposed an optimized point-cloud compression scheme to enhance the effectiveness of the point-cloud compression.

The structure of the paper is organized as follows. We first review the state of point-cloud compression based on the convolutional neural network in Section 2. Then, we describe our working methods in detail in Section 3, followed by the results and the discussions in Section 4. Finally, we conclude the work in Section 5.

## 2. Related Work

The goal of point-cloud compression is to achieve a balance between high compression ratio, low distortion rate, and computing cost. The MPEG-3DG working group divided point-cloud compression standards into video-based point-cloud compression (V-PCC) and geometry-based point-cloud compression (G-PCC) according to processing methods. V-PCC aims to provide low-complexity decoding capability for applications requiring real-time decoding, such as virtual/augmented reality and immersive communication [14]. G-PCC is believed to provide efficient lossless and lossy compression for the deployment of autonomous driving, 3D maps, and other applications using point clouds generated by radar [15].

Aiming at the traditional point-cloud compression method based on octree, Diogo et al. proposed geometric octree coding of a point cloud based on an intra frame context [16,17]. The octree structure provides better context for entropy coding and improves the average rate. An algorithm for motion estimation and compensation was presented by Thanou et al. [18]. It is utilized to eliminate the temporal redundancy from the predictive coding of 3D positions and the color properties of point-cloud sequences. On the basis of this, Quach et al. suggested a novel motion-compensated approach to encoding dynamic voxelized point clouds at low bit rates where both the geometry and the color are encoded with distortion, allowing for reduced bit-rates [19]. Mekur et al. implemented point-cloud coding and decoding by dividing the octree voxel space into multiple macroblocks that are progressively subdivided to achieve intra-frame coding, mainly applied for 3D immersive video for general purpose and real-time time variation to improve point cloud acquisition rate to a large extent [20]. However, the octree-based compression technique tends to create “blocky” outputs at low to medium bit rate rendering stages and causes an exponential drop in the number of point clouds when the depth of the tree is decreased.

In recent years, due to the development and breakthrough of deep learning in the field of image research, Wu et al. represented a geometric 3D shape as a probability distribution of binary variables on a 3D voxel grid, using a convolutional deep neural network [21]. Valenzise et al. found that compression approaches based on deep auto-encoders can achieve coding performance higher than JPEG 2000 [22]. Huang et al. proposed a 3D point-cloud geometric compression method based on deep learning, which was an auto-encoder that also supported the parallel compression of multiple models via the GPU, considerably increasing processing efficiency [23]. When compared to PCL compression [24] and Draco compression [25], this method retains the original shape with little loss. To learn how to represent disordered point clouds, Panos et al. constructed an auto-encoder using convolutional layers and fully linked layers [26]. They compared the generative capacities of several distinct autoencoder-based GANs that were trained as generative networks [27]. The approach of compressing a point cloud using convolution was improved by Ball et al., who also suggested utilizing additive uniform noise in place of quantization during training and doing actual quantization during evaluation [28]. Inspired by this, Maurice et al. proposed a convolution transformation learning method for lossy point cloud geometric compression [29]. They also directly learned filters from data, taking into account quantization and rate-distortion (RD) in training. In contrast to the octree-based method, this method’s model has strong universality and does not exhibit an exponential decline in the number of points when the bit rate is decreased.

In addition, deep learning-based convolutional neural networks showed promising performance in medical detection, GPS, and risk assessment [30,31,32]. Recent research on binary neural networks (BNNs) has also been successively published. Liu et al. introduced a reshaped point-wise convolution (RPC) to replace the conventional one to build binary neural networks (BNNs) [33]. On the other hand, Xu et al. first attempted to optimize BNNs from a bilinear perspective, which improved the learning process of BNNs by correlating intrinsic bilinear variables during backward propagation [34]. Similarly, Jin et al. introduced a deep-walk strategy into graph convolutional networks (GCNs) to efficiently explore the global graph information, which enabled the more efficient extraction of potential representations of graph structure data [35]. Moreover, Zeng et al. proposed a face neural volume rendering (FNeVR) network and lightweight pose editor to enhance the facial details for image rendering [36].

Combined with the above research, our optimized convolutional neural network framework greatly improves the quality of point-cloud compression, and the work is highly scalable, which is an important reference for related work in this area.

## 3. Methods

**Figure 1:**
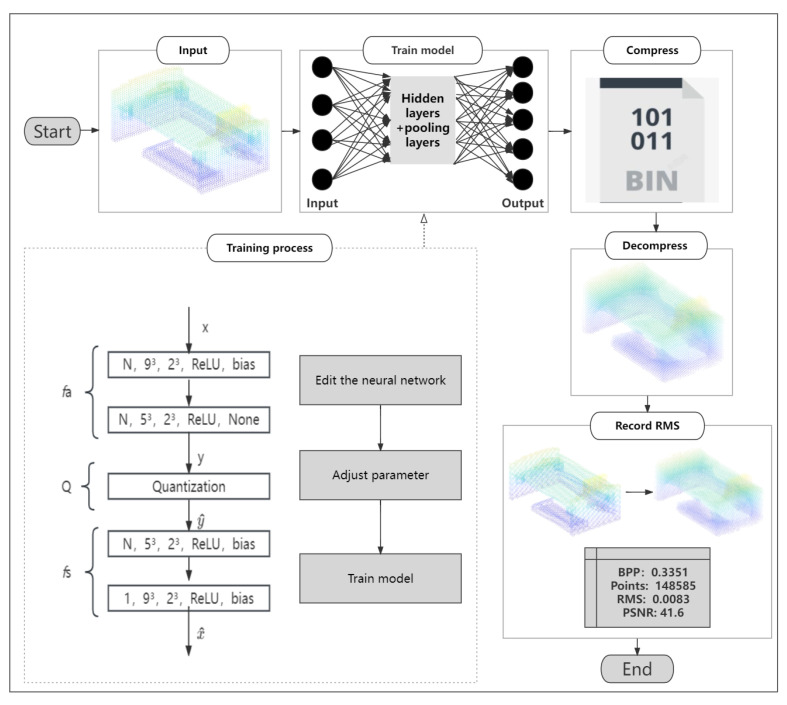
Flow chart of CNN-based 3D point-cloud compression scheme. First, we input the point-cloud model and then use the trained model to obtain the compressed binary file. Then, we decompress the compressed file to compare the decompressed point-cloud model with the one before decompression. Finally, we evaluate the performance by recording the RMS and other parameters between them. The steps of training model are as follows (dash line): First, edit the neural network mesh and adjust convolutional neural network depth, stride, and activation function. Second, adjust the compression accuracy and the number of training rounds. Third, observe model training index total loss and total quantities. If the training requirements are not fulfilled, the model will be retrained until it meets the requirements.

Figure 1 shows flow chart of CNN-based 3D point cloud compression scheme. The remainder of the section is organized as follows. We first specify the model-training process in Section 3.1. Then, we describe the experimental process of obtaining the outputs of our network in Section 3.2. Finally, We explain the loss-calculation process in Section 3.3.

### 3.1. Model Training

#### 3.1.1. Neural Network Structure

The first step in this work is to design the initial structure of the convolutional neural network. In our method, *x* represents the input original point cloud, $\hat{x}$ represents the output decompressed point cloud, and $f_{a}$ represents the analysis transformation. Furthermore, $y=f_{a}\left(x\right)$, *Q* is the quantification function, y=Q(y), $f_{s}$ is the synthesis transformation, $\hat{x}=f_{s}\left(\hat{y}\right)$. We use *N* to represent the filter, and $s^{3}$ and $p^{3}$ to represent padding and stride. For example, $9^{3}$ and $2^{3}$ represent 9×9×9 padding, and the filter stride is 2×2×2.

**Figure 2:**
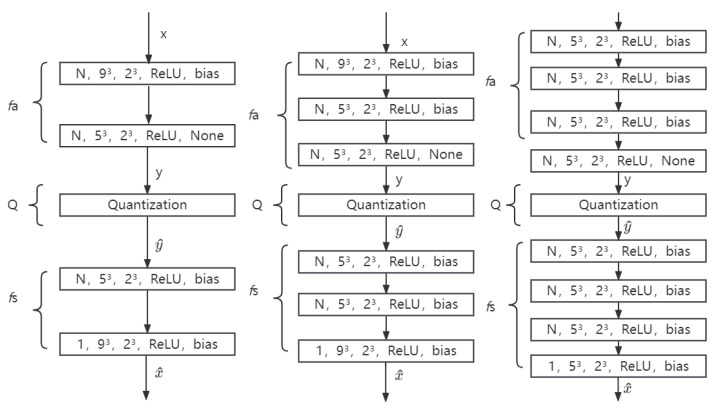
The architecture of the used neural network with different layers of 2 strides. Layers are specified using the following format: the number of feature maps, filter size, strides, activation, and bias. *Relu* in the figure is the reference activation function. Left to right shows 2 layers, 3 layers, and 4 layers with 2 strides, respectively.

**Figure 3:**
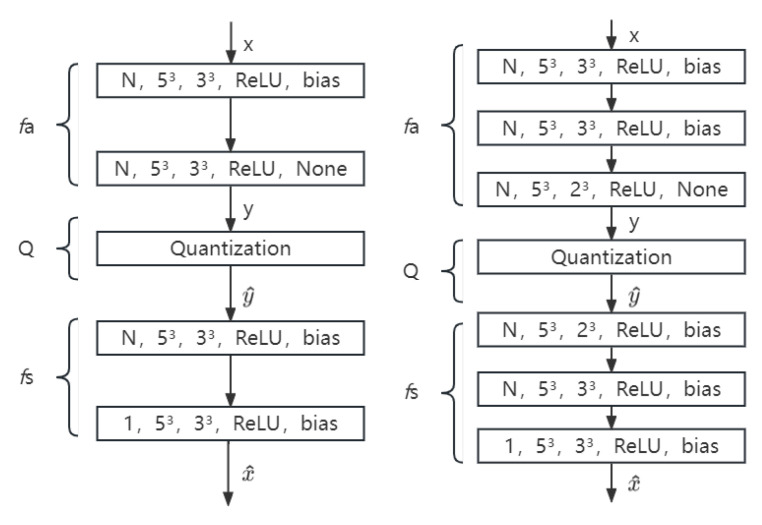
The architecture of the used neural network with different layers of 3 strides. *Relu* in the figure is the reference activation function. Left to right shows 2 layers and 3 layers with 3 strides, respectively.

We use 2 layers, 3 layers, 4 layers with 2 strides and 2 layers, and 3 layers with 3 strides for the test. The architecture of the used neural network is shown in Figure 2 and Figure 3. The minimal compression requirements cannot be achieved by 4 layers with 3 strides, and thus it is not taken into account in this case.

#### 3.1.2. Activation Functions

The choices of activation functions have a significant impact on the convolutional neural network. An activation function is a function transformation between the input and output of the neural network layer, which allows one to add nonlinear factors and enhance the expression ability of the model. However, in practical applications, it remains challenging to determine which activation function is the best or the most effective. In this study, we evaluate nine activation functions.

(1) *Sigmoid*.
  The *Sigmoid* function is also known as the logistic function, which is used for the output of hidden layer neurons and the binary classification. The value range is (0, 1), which can map a real number to the interval of (0, 1). The effect is more preferable when the feature difference is complex or not large.
  (1) $$f\left(x\right)=\frac{1}{1+e^{-x}}$$
(2) *Tanh*.
  The *Tanh* activation function is also named as the hyperbolic tangent activation function. Similar to the *Sigmoid* function, the *Tanh* function also uses the truth values, and the *Tanh* function converts it to the range of −1 to 1. Therefore, the output of the *Tanh* function is zero-centered, which solves the problem of slow convergence of *Sigmoid* function and improves the convergence speed compared with *Sigmoid*.
  (2) $$\begin{array}{c} Tanh(x)=\frac{e^{x}-e^{-x}}{e^{x}+e^{-x^{\prime }}}\\ Tanh(x)=2Sigmoid(2x)-1 \end{array}$$
(3) *Relu*.
  The *Relu* function converts the output of some neurons to be 0, which causes the sparsity of the network. Thus, the *Relu* function has the advantages of reducing the parameter’s interdependence and alleviating the occurrence of over-fitting problems.
  (3) Relu(x)=max(0,x)
(4) *Leaky Relu*.
  Compared to the *Relu* function, the output of this function is no longer 0 under the condition that *x* is negative. Here, *a* is usually taken as 0.01. Thus, it solves the problem of gradient disappearance in the *Relu* activation function, which also shows the advantages of efficient computation and faster convergence rates than the *Sigmoid*/*Tanh* function.
  (4) $$Leaky\;Relu(x)=\left\{\begin{array}{c} x(x\geq 0)\\ \mathrm{ax}(x<0) \end{array}\right.$$
(5) *Elu*
  The exponential linear unit (*Elu*) activation function is proved to have high noise robustness and can convert the average activation value of neurons that approach zero. Due to the necessity to calculate the index, the calculation cost is high.
  (5) $$Elu(x)=\left\{\begin{array}{cc} x, & \;\mathrm{if}\;x>0\\ \alpha \left(e^{x}-1\right), & \;\mathrm{otherwise}\; \end{array}\right.$$
(6) *Gelu*
  The Gaussian error linear units (*Gelu*) function introduces the idea of stochastic regularity in activation, a probabilistic description of neuronal input that intuitively complies with our natural understanding.
  (6) $$Gelu\left(x\right)=\frac{x}{1+e^{-1.702x}}$$
(7) *Selu*
  The scaled empirical linear units (*Selu*) function introduces the attribute of self-normalization, which avoids the problem of sudden disappearance or the explosive growth of the gradient. Therefore, this function enables the learning process to be more stable than other functions mentioned above.
  (7) $$\begin{array}{c} Selu(x)=\lambda \left\{\begin{array}{cc} x & \;\mathrm{if}\;x>0\\ \alpha e^{x}-\alpha & \;\mathrm{if}\;x\leq 0 \end{array}\right. \end{array}$$
(8) *R-Relu*
  The Random Relu (*R-Relu*) function is also a variant of *Leaky Relu*, where the slope of negative value is random in training.
  (8) $$\begin{array}{c} y_{ji}=\left\{\begin{array}{cc} x_{ji} & \;\mathrm{if}\;x_{ji}\geq 0\\ a_{ji}x_{ji} & \;\mathrm{if}\;x_{ji}<0 \end{array}\right.\\ \;\mathrm{where}\;\\ a_{ji}∼U\left(l,u\right),l<u\;\mathrm{and}\;l,u\in \left[0,1\right) \end{array}$$
(9) *HardSwitch*
  The *HardSwitch* activation function is used to approximate the *Switch* activation function. This function introduces subsection calculation, which greatly reduces the amount of calculation under the condition. This allows one to retain the feature, and thus the *Switch* function can enable the neural network layer to have more expressive ability.
  (9) $$HardSwish(x)=x\cdot \left\{\begin{array}{cc} 1, & x\geq 3\\ \frac{x}{6}+\frac{1}{2}, & -3<x<3\\ 0, & x\leq -3 \end{array}\right.$$

#### 3.1.3. Training

In this work, the model date we use is from the Princeton 3D Object Dataset ModelNet40, which provides a comprehensive clean collection of 3D CAD models for objects [21,37]. ModelNet40 is collection of 3D CAD models for object classification and segmentation, including 40 categories, 9843 (80%)point-cloud data in the training set, and 2468 (20%) point-cloud data in the testing set. The experimental environment is configured with Python = 3.6 and Tensorflow = 0.12. The configuration of the convolution neural network is filters N = 32, batch size = 64, and Adam’s learning rate $lr=10^{-4}$.

In our work, we use two measurement indicators in the training process, total_loss and total_quantiles_loss. If total_loss<2 and total_quantiles_loss<50, we can determine that we have obtained an effective model. Before the training starts, we need to set the training rounds. According to the experience gained in the experiments, the general setting is 350–400 rounds. It worth to mention that more training rounds is not always better because too many training rounds cannot increase the time cost and may even reduce the quality of the model of compressed 3D point cloud. If the experimental results do not meet the requirements, we need to restart the training process until we obtain the experimental model that meets the requirements.

### 3.2. Network Outputs

First, we input the point-cloud model and then use the trained model to obtain the compressed binary file. We compute the size of the compressed file *b* as follows:

(10) BPP=b∗8/n,

where BPP represents bits per pixel, *n* represents the number of points in the point cloud file, and a lower BPP means a higher compression rate. Then, we decompress the compressed file to obtain a reconstructed 3D point-cloud model and compare it with original model. Furthermore, we use the point cloud library (PCL) to measure the reconstruction error between the original input point cloud and the reconstructed data.

### 3.3. Loss Function

Our decoding procedure is viewed as a binary classification problem in which the existence or absence of the voxel grid’s point *z* is determined. We resolve the decompressed point cloud $\hat{x}={\hat{v}}_{S}$ into its individual voxel *z*, which is related to the value of $p_{z}$. Most voxels are not occupied as a result of the point cloud’s sparse distribution, so the majority of its $v_{S}\left(z\right)$ values are zero. To balance the empty voxels and occupied voxels, we use

(11) $$FL\left(p_{z}^{t}\right)=-\alpha_{z}{\left(1-p_{z}^{t}\right)}^{\gamma }\log\left(p_{z}^{t}\right)$$

if $v_{S}\left(z\right)=1$, $p_{z}^{t}$ is equal to $p_{z}$ or $1-p_{z}$. The compressed point cloud’s focal loss is indicated as:

(12) $$FL\left(\tilde{x}\right)=\sum_{z\in S}FL\left(p_{z}^{t}\right)$$

Our final loss can be expressed as L=λD+R, with *D* denoting the distortion derived from focal loss and *R* denoting the quantity of bits per input occupied voxel.

## 4. Result and Discussion

We use the evaluation criteria released by the moving picture experts group (MPEG) [38] and choose the symmetric rms distance between the point clouds (RMS) to evaluate the compression performance of point cloud. The point cloud is made up of a collection of points denoted by (x,y,z) and a number of attributes, with color components (y,u,v) playing a crucial role. We can define the point *v*, which has a specific position in a 3D space (x,y,z) and an optional colour attribute *c*, where the components of the attribute *c* are r,g,b or y,u,v and optional alternative parameters may represent normal or texture maps.

(13) $$\;\mathrm{point}\;v=\left(\left(\left(x,y,z\right),\left[c\right],\left[a_{0}\ldots a_{A}\right]\right):x,y,z\in R,\left[c\in \left(r,g,b\right)|r,g,b\in N\right],\left[a_{i}\in \left[0,1\right]\right]\right)$$

At present, the point cloud consists of only a collection of *K* loosely ordered points:

(14) $$\;\mathrm{Original}\;\mathrm{Point}\;\mathrm{Cloud}\;V_{\mathrm{or}\;}=\left\{\left(v_{i}\right):i=0\ldots .K-1\right\}$$

The first cloud serve as the standard for judging the quality of the second inferior cloud $V_{deg}$ that consists of *N* points.

(15) $$\;\mathrm{Degraded}\;\mathrm{Point}\;\mathrm{Cloud}\;V_{\deg\;}=\left\{\left(v_{i}\right):i=0\ldots ..N-1\right\}$$

RMS can be calculated as follows:

(16) $$d_{rms}\left(V_{or},V_{deg}\right)=\sqrt{\frac{1}{K}\sum_{vo\in Vor}{\left[vo-vd_{-}\text{nearest-neighbour}\right]}^{2}}$$

(17) $$d_{\text{symmetric--rms}\;}\left(V_{or},V_{\deg}\right)=\max\left(d_{rms}\left(V_{or},V_{\deg\;}\right),d_{rms}\left(V_{\deg},V_{or}\right)\right)$$

The smaller the RMS value will result in less error between the original point cloud and the decompressed point cloud, so the point cloud will be more effectively compressed.

### 4.1. Evaluation of the Compression by Different Depth of The Network

Different depths determine the shape of the output graph. By deepening the network, we can decompose the problems to be studied in different levels. At present, in the study of depth, it is generally believed that if the problem is more complex and higher order, it needs a deeper network. By deepening the depth of the convolution neural network, the better the sensitivity and convergence of the model can be improved. In practice, it is not the deeper the better because the deeper the structure, the more likely the gradient disappearance phenomenon happens. Meanwhile, the output of each layer will lose part of the edge information. In our experiment, the depth less than 4 in the network can obtain better experimental results.

To ensure the effectiveness of the experiment, we first verify the framework in three layers and two strides using activation functions of *Relu*, *Selu*, and *Elu*.

**Figure 4:**
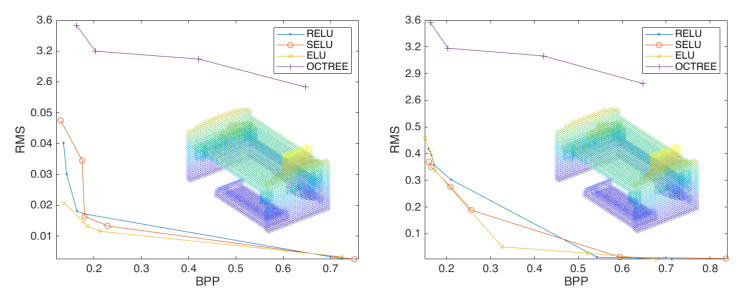
The compression performance of the scheme configured with three layers of convolution and two strides (**left**) and two layers convolution and two strides (**right**), using the activation functions *Relu*, *Selu*, and *Elu*.

**Figure 5:**
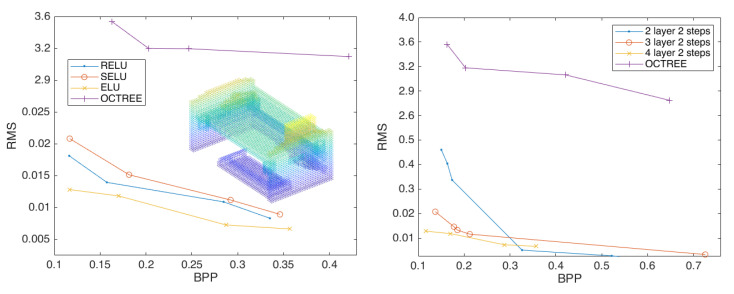
The compression performance of the scheme configured with four layers of convolution, and two strides, using the activation functions *Relu*, *Selu*, and *Elu* (**left**). Comparison of convolution effects of each layer (**right**).

Figure 4 demonstrate that our approach is successful and efficient, fulfills the anticipated demands, and outperforms octree compression by a wide margin. Then, we expand the experiment and change the depth of the convolutional neural network to two and four layers to observe the impact on the point-cloud compression effect. The results are shown in Figure 5.

By comparing the best performing curves in each plot in Figure 5, we find that the effect of convolutional neural network compression point cloud is better than that of octree compression, the compression effect of three layers of compression convolution is better than that of two layers compression convolution, and the compression effect of four layers of compression convolution is better than that of three layers compression convolution, while using the activation function *Elu*, which is also the best compression effect. Specifically, the four layer compression convolution is better than that of the three layer compression convolution, with an average compression rate that has been improved by 32.29%.

### 4.2. Evaluation of the Compression by Different Number of Strides

The purpose of setting the stride is to reduce the number of input parameters and reduce the amount of calculation. The value of the side parameter is the multiple of reduction. In general, the smaller the stride size is, the easier it is to obtain local optimization. Additionally, the larger the stride size is, the better it will obtain global optimization. However, too large of a step size will lose a lot of graphic details. When preparing for our experiment, we found that if the stride is set to 1, the program would be down due to excessive computation. If the step size is more than 3, the compression effect is significantly reduced. For this reason, we chose strides 2 and 3 as the experimental parameters and used the half-filling method to increase the size of the output graphics.

**Figure 6:**
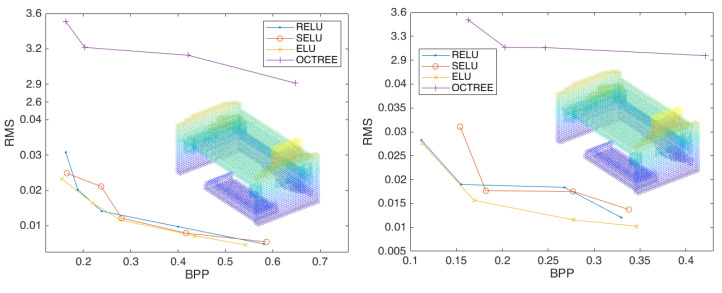
The compression performance of the scheme configured with two layers of convolution and three strides (**left**) and three layers of convolution and three strides (**right**), using the activation functions *Relu*, *Selu*, and *Elu*.

**Figure A1:**
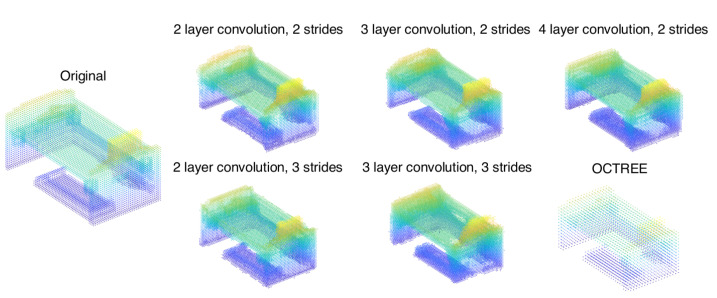
The compression performance of the scheme configured with different depths and strides.

In order to further explore the impact of stride on the point-cloud compression effect, we changed the experimental stride to 3 and repeated the above experiment. The results showed that the compression with four layers and three strides did not meet the experimental requirements, which is excluded here. Figure A1 shows the compression performance of the scheme configured with different depths and strides. Figure 6 shows the overall compression performance with different specifications to the networks.

**Figure 7:**
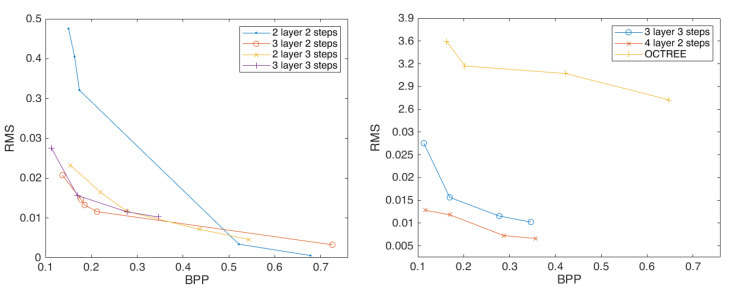
Comparison of stride effects of each layer (**left**); curve comparison for best results (**right**).

Figure 7 demonstrates that when the depth of compression convolutions are the same, with three layers of compression convolution, the compression effect of three strides is significantly better than that of two strides. This shows that the compression impact of two layers of compression convolution is less effective under two strides condition. Under three layers of compression convolution using the activation function *Elu*, the compression effect of two strides is better than that of stride three, with an average compression rate improved by 28.35%. This demonstrated that by changing the stride one can optimize the compression effect of the point cloud to a certain extent, but it is unstable.

Furthermore, we have discovered that the compression effect produced by four layers and two strides is the best. Meanwhile, the compression impact of three strides is discovered to not always be worse than that of two strides. Throughout all of the experiments, the activation function *Elu* exhibits the best compression result, demonstrating the stability of the activation function’s support for point-cloud compression.

### 4.3. Evaluation of the Compression of Different Activation Functions

The activation functions are significantly important in neural networks. They determine whether a neuron is activated, whether the information received by the neuron is useful, and whether it should be left or abandoned. The neural network without an activation function is a linear regression model. In the research field of graphics processing, the nonlinear transformation of the activation function can make the neural network process very complex. The application scenarios of different activation functions are also different. Although their introductions have a certain reference significance for us to select activation functions, for different studies, the activation functions that are most suitable for the model can only be determined based on experimental comparison.

**Figure A2:**
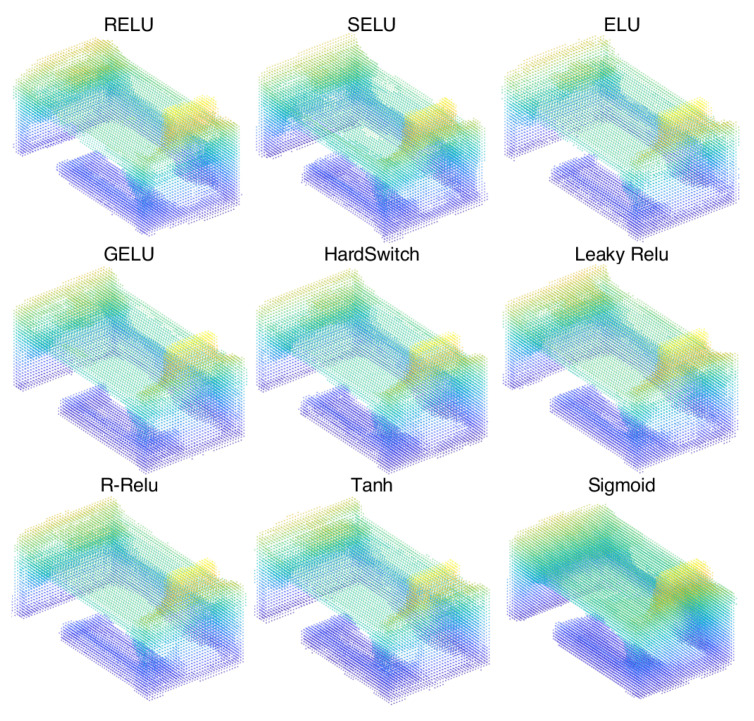
The compression effects of 3D point clouds with different activation functions.

Therefore, we continue to investigate the activation function and provide the following six activation functions for experiments: *Sigmoid*, *Tanh*, *Leaky Relu*, *R-Relu*, *Gelu and HardSwitch*. Figure A2 shows the compression effects of 3D point clouds with different activation functions.

**Figure 8:**
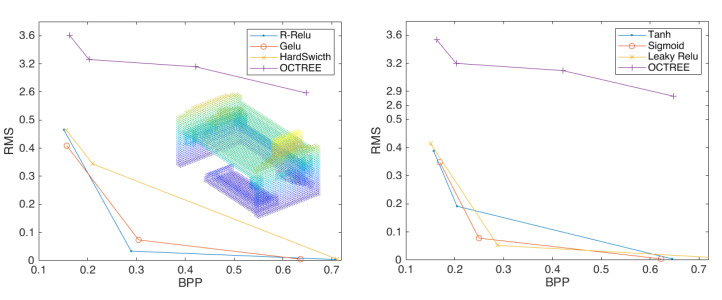
The compression performance of the scheme configured with two layers of convolution and two strides, using the activation functions *R-Relu*, *Gelu*, and *HardSwitch* (**left**) and *Tanh*, *Sigmoid*, and *Leaky Relu* (**right**).

**Figure 9:**
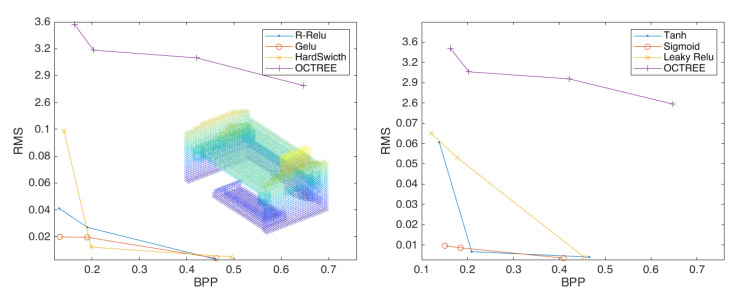
The compression performance of the scheme configured with three layers of convolution and two strides using the activation functions *R-Relu*, *Gelu*, and *HardSwitch* (**left**) and *Tanh*, Sigmoid, and Leaky Relu (**right**).

**Figure 10:**
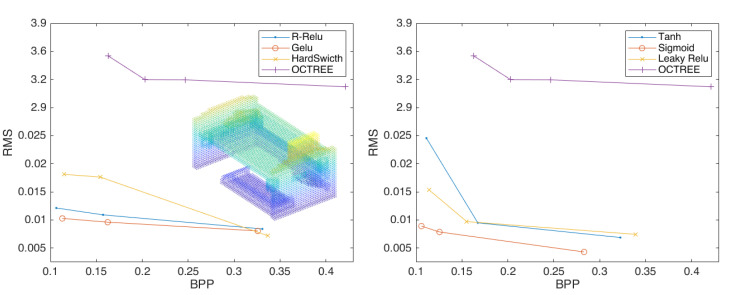
The compression performance of the scheme configured with four layers of convolution and two strides using the activation functions *R-Relu*, *Gelu*, and *HardSwitch* (**left**) and *Tanh*, *Sigmoid*, and *Leaky Relu* (**right**).

**Figure 11:**
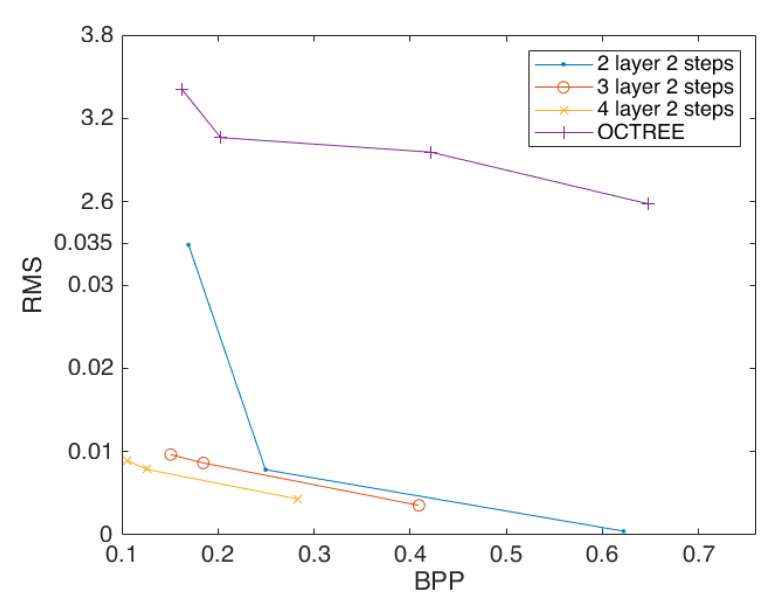
Three curves that perform best at each depth using the *Sigmoid* function.

Figure 8 shows that when the depth is 2, the best activation function is *Sigmoid*, which is not far from the other functions. Figure 9 shows that when the depth is 3, the *Sigmoid* function is significantly improved compared to the other functions. Figure 10 and Table 1 shows that the *Sigmoid* function can still achieve better a compression effect when the depth is 4. In Figure 11, the highest performance across all experiments is demonstrated by the point-cloud compression utilizing the *Sigmoid* function of four layers and two strides. Therefore, the *Sigmoid* function performs best at improving the compression effect of point clouds.

## 5. Conclusions

**Figure 12:**
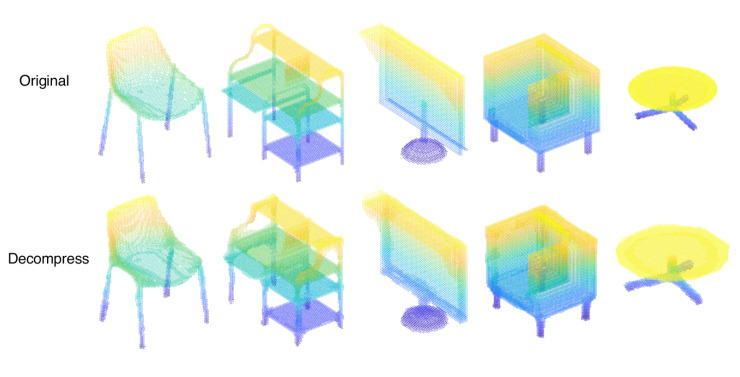
Results from different point clouds.

In this work, we have used the optimized convolutional neural network to compress the point cloud and have investigated the compression effect of the point cloud at the same time. By varying the depth, strides, and activation functions of various convolutional neural networks, we have evaluated the impact on the point-cloud compression effect and further the research that has been done so far on compressing point clouds using convolutional neural networks. We have found that the deeper the depth of the neural network is, the better the compression effect of the point cloud is. More specifically, it can significantly improve the compression effect of the point cloud when the depth of the convolutional neural network is 4. Meanwhile, the compression effect of the point cloud is also affected by the stride; the shorter the stride size achieves the better experimental effect. Since an excessively small stride can lead to a significant increase in computational costs, the most suitable step size for our experiments is 2. We have discovered that the improvement of the point-cloud compression effect by changing the activation function is stable. Furthermore, We have concluded that the *Sigmoid* function outperformed the other activation functions mentioned above by comparing the ultimate compression effects. Therefore, we obtain the optimal parameter configuration of four layers and two strides using the *Sigmoid* activation function, which is 208.56% higher than the original framework proposal’s default configuration of three layers of convolution and two strides using the *Relu* function. We have applied our experimental scheme to other 3D models, which demonstrated the validity and generality of our experimental results in Figure 12.

Based on this study, as a part of future works, it is possible to further improve the compression performance by changing the data set used for training, alternating other activation functions not involved in other articles, changing the depth of the convolution network used, etc. In addition, the time cost is also one of the factors used to evaluate the compression approaches. In this work, we have not taken the time cost into account because the model training takes a relatively short period of time.

## Figures and Tables

**Table 1:** RMS of 9 activation functions with four layers of convolution and two strides. Italic indicates the activation function, and bolded indicates the minimum RMS data.

	Accuracy	$5\times 10^{-5}$	$1\times 10^{-5}$	$5\times 10^{-6}$
Function				
*Relu*		0.00831488	0.0139485	0.0181215
*Selu*		0.00893118	0.0151461	0.0208207
*Elu*		0.00664042	0.0118255	0.0128144
*Tanh*		0.00688373	0.0094999	0.0245558
*Leaky Relu*		0.00744641	0.0097154	0.0153705
*R-Relu*		0.00840702	0.0109112	0.0121359
*Gelu*		0.00805524	0.0096145	0.0102793
*HardSwitch*		0.00719350	0.0176457	0.0181156
*sigmoid*		**0.00431234**	**0.0078694**	**0.0089128**

## Data Availability

The common dataset ModelNet40 used in this study can be obtained from link https://modelnet.cs.princeton.edu/.
